# Lung Ultrasound-Guided Surfactant Therapy in Neonatal Pneumothorax and Pulmonary Hemorrhage: Pathophysiology, Diagnostic Ultrasonography, and Emerging Clinical Approaches

**DOI:** 10.3390/children13060784

**Published:** 2026-06-04

**Authors:** Adina Mihaela Frenti, Florin Filip, Elena Tătăranu, Vlad Dima, Roxana Axinte, Alina Sânzâiana Melinte, Mirabela Dima, Iulia Ciubotariu, Petronela Vicoveanu, Smaranda-Ileana Jurchis-Irimie, Smaranda Diaconescu

**Affiliations:** 1 Doctoral School, University of Medicine, Pharmacy, Sciences and Technology “George Emil Palade”, 540142 Targu Mures, Romania; 2 Clinical Department of Neonatology, Sf. Ioan cel Nou Emergency Hospital, 720224 Suceava, Romania; 3 Department of Pediatric Surgery, Sf. Ioan cel Nou Emergency Hospital, 720224 Suceava, Romania; 4 Faculty of Medicine and Biological Sciences, Ștefan cel Mare University of Suceava, 720229 Suceava, Romania; 5 Clinical Department of Pediatrics, Sf. Ioan cel Nou Emergency Hospital, 720224 Suceava, Romania; 6 Department of Neonatology, Filantropia Clinical Hospital, 011132 Bucharest, Romania; 7 Faculty of Medicine, Carol Davila University of Medicine and Pharmacy, 050474 Bucharest, Romania; 8 Department of Puericulture and Neonatology, Victor Babeș University of Medicine and Pharmacy, 300041 Timisoara, Romania; 9 Department of Neonatology, Clinical County Emergency Hospital No. 1 Timisoara, 300098 Timisoara, Romania; 10 Department of Obstetrics and Gynecology, Sf. Ioan cel Nou Emergency Hospital, 720224 Suceava, Romania; 11 Doctoral School of Medicine, University of Oradea, 410073 Oradea, Romania; jurchis.smaranda@spjsv.ro; 12 Department of Physical and Rehabilitation Medicine, University of Oradea, 410073 Oradea, Romania; 13 Department of Physical and Rehabilitation Medicine, Clinical County Emergency Hospital “Dr. Gavril Curteanu”, 410157 Oradea, Romania; 14 Faculty of Medicine, University Titu Maiorescu, 040441 Bucharest, Romania

**Keywords:** lung ultrasound, point-of-care ultrasound, POCUS, surfactant therapy, neonatal pneumothorax, pulmonary hemorrhage, respiratory distress syndrome, neonatal imaging, LUS score, minimally invasive surfactant administration

## Abstract

Background and Objectives: Lung ultrasound (LUS) has fundamentally transformed neonatal respiratory diagnostics, offering a radiation-free, bedside-applicable modality capable of guiding surfactant therapy, characterizing pulmonary pathology, and monitoring treatment response in real time. While surfactant replacement therapy is firmly established for neonatal respiratory distress syndrome (RDS), its role in acute complications—specifically pulmonary hemorrhage (PH) and pneumothorax (PTX)—remains uncertain and heterogeneous in clinical practice. This review examines how LUS-based phenotyping can improve the diagnostic precision and therapeutic sequencing of surfactant administration in these high-risk scenarios, and how comorbidities such as hemodynamically significant patent ductus arteriosus, persistent pulmonary hypertension, sepsis, and coagulopathy modulate clinical outcomes. Materials and Methods: We conducted a structured narrative review of studies published from 2020 onward, sourced from PubMed, Web of Science, Semantic Scholar, and Mendeley, using PRISMA-inspired selection principles. The search combined terms including “lung ultrasound,” “neonatal POCUS,” “surfactant therapy,” “pulmonary hemorrhage,” “neonatal pneumothorax,” and “LUS score.” Studies focusing on neonatal populations, clinical LUS applications, and surfactant use in PH and PTX were prioritized. Results: Quantitative LUS scoring systems (range 0–18) predict surfactant need and re-dosing with AUC values of 0.85–0.87, outperforming clinical estimates alone. In PH, LUS reveals dense consolidation with alveolar flooding patterns, guiding the timing of rescue surfactant after hemodynamic stabilization; response monitoring via serial LUS is feasible and informative. In PTX, hallmark signs—absent lung sliding, loss of B-lines, and the pathognomonic lung point—allow diagnosis within seconds, guiding immediate thoracentesis and subsequent surfactant administration if underlying RDS is confirmed. Nationally implemented LUS protocols in neonatal intensive care units have demonstrated significant reductions in radiation exposure without compromising diagnostic accuracy. Conclusions: LUS-guided decision algorithms—integrating ultrasonographic phenotyping, quantitative scoring, and hemodynamic assessment—represent the current best framework for individualizing surfactant therapy in neonatal PH and PTX. Standardization of POCUS training and protocol implementation in neonatal units is essential. Prospective multicenter trials are urgently needed to define optimal indications, timing, and dosing in these vulnerable populations.

## 1. Introduction

Neonatal respiratory failure remains one of the most pressing challenges in perinatal medicine worldwide. Approximately 13.4 million infants are born preterm annually—roughly one in ten live births—and respiratory distress syndrome (RDS) accounts for a disproportionate share of neonatal mortality, particularly in low- and middle-income countries where nearly 45% of prematurity-related deaths are attributable to lung immaturity [1,2]. Surfactant replacement therapy and continuous positive airway pressure (CPAP) have transformed outcomes in this population, reducing mortality and the severity of respiratory morbidity across gestational ages [3,4,5].

Beyond RDS, a subset of critically ill neonates develops acute complications—most notably pulmonary hemorrhage (PH) and pneumothorax (PTX)—that challenge even experienced neonatal teams. These conditions share a common need for rapid, accurate diagnosis and individualized therapeutic response, yet neither has been the subject of large, definitive randomized trials regarding surfactant use. Clinical decision-making therefore depends heavily on pathophysiological reasoning, bedside assessment, and increasingly, real-time imaging [6,7].

Over the past two decades, lung ultrasound (LUS) has undergone a quiet revolution in neonatal medicine. From its origins in adult critical care through the pioneering work of Lichtenstein, neonatal LUS has evolved into an indispensable point-of-care tool, endorsed by international expert consensus bodies including the European Society of Paediatric and Neonatal Intensive Care (ESPNIC) [8]. The neonatal lung—with its high water content, compliant chest wall, and immature rib ossification—is acoustically ideal for ultrasonographic interrogation, yielding high-resolution images at standard probe frequencies without ionizing radiation [9,10].

In this context, the integration of LUS into surfactant decision pathways represents a paradigm shift: from empirical, FiO_2_-threshold-based protocols toward phenotype-driven, ultrasonographically-informed algorithms. Quantitative LUS scoring systems have demonstrated predictive accuracy for surfactant need (AUC 0.87) and re-dosing decisions (AUC 0.854) that rival or exceed traditional clinical criteria [11,12]. Delivery-room LUS protocols now provide actionable respiratory transition data within the first minutes of life [9], while quality improvement programs in neonatal intensive care units (NICUs) have shown that systematic LUS implementation reduces chest radiograph use by over 20%, without compromising clinical accuracy [10].

This review synthesizes current evidence on surfactant administration in neonatal PH and PTX through a deliberate ultrasonographic lens. We examine the pathophysiology of surfactant dysfunction in each condition, the diagnostic role of LUS as a primary imaging tool, validated scoring systems and their clinical cut-offs, the influence of hemodynamic comorbidities on therapeutic response, and the emerging evidence base for minimally invasive surfactant administration (LISA) in complex neonatal scenarios. Our objective is to offer a clinically actionable, LUS-anchored framework for decision-making in these high-risk neonatal populations [6,13].

## 2. Materials and Methods

**Figure 1:**
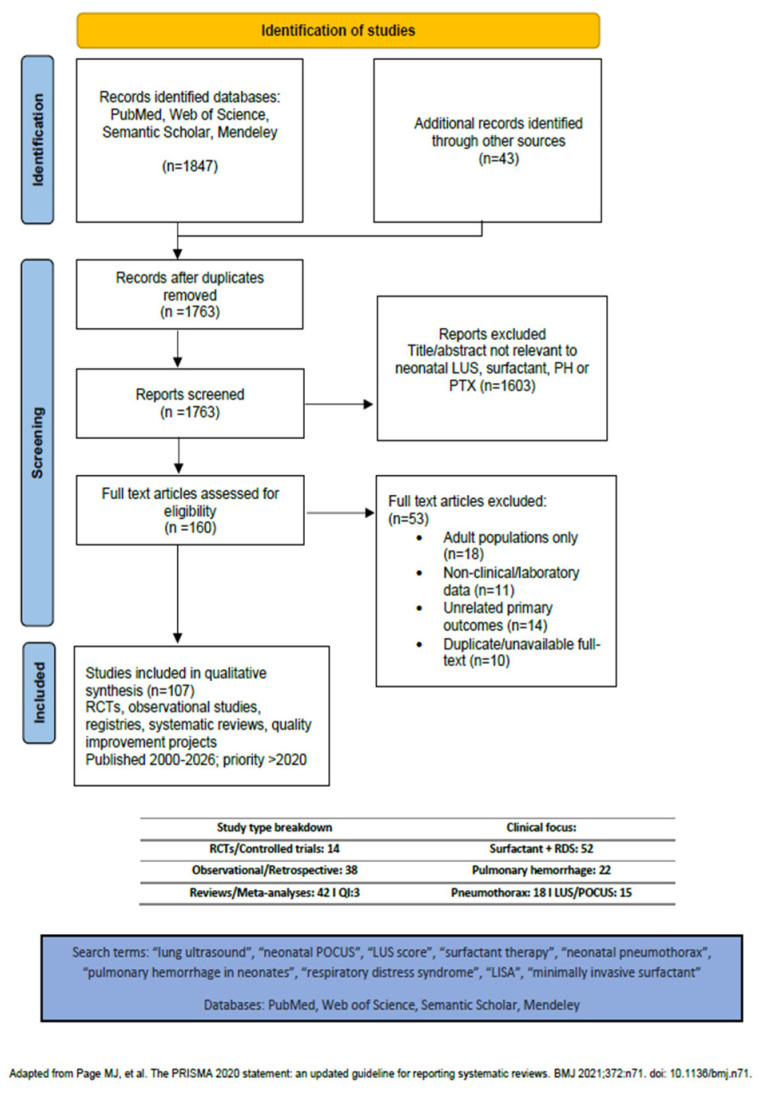
PRISMA flow diagram illustrating the study selection process for this narrative review. Databases searched: PubMed, Web of Science, Semantic Scholar, Mendeley. Total records identified: n = 1847 (databases) + 43 (other sources). After removal of duplicates, title/abstract screening, and full-text assessment for eligibility, 107 studies were included in the qualitative synthesis [14].

We conducted a structured narrative literature review of studies published from 2020 onward, examining the role of LUS and surfactant therapy in neonatal PTX and PH. Electronic databases including PubMed, Web of Science, Semantic Scholar, and Mendeley were systematically searched. The search strategy combined the following terms using Boolean operators: “lung ultrasound,” “neonatal POCUS,” “LUS score,” “surfactant therapy,” “neonatal pneumothorax,” “pulmonary hemorrhage in neonates,” “respiratory distress syndrome,” “minimally invasive surfactant administration,” and “LISA.” Although this is not a systematic review, selection was guided by PRISMA-inspired principles to enhance transparency and reproducibility (Figure 1).

The literature search was conducted between November 2025 and March 2026 and included studies published from 1 January 2000 to 30 March 2026; study selection and screening were independently performed by A.M.F., V.D., and S.D., with disagreements resolved through discussion.

Studies were screened based on relevance to neonatal populations, clinical application of LUS and/or surfactant in PTX and PH, and quality of evidence. Original research articles, clinical trials, observational studies, national registry analyses, quality improvement projects, and high-quality reviews were included. Studies restricted to adult populations, non-clinical data, or unrelated outcomes were excluded. Priority was given to studies reporting quantitative LUS parameters, validated scoring systems, and patient-level outcome data.

## 3. Results

The results are organized around five interconnected clinical domains: (3.1) lung ultrasound as a primary diagnostic tool in neonatal respiratory failure; (3.2) pulmonary hemorrhage—epidemiology, ultrasonographic features, and surfactant rationale; (3.3) pneumothorax—LUS-based diagnosis, risk stratification, and management; (3.4) pulmonary surfactant—biology, preparations, and dosing algorithms; and (3.5) LUS-guided surfactant strategies—scoring systems, administration techniques, and comorbidity impact. Across all domains, ultrasonographic phenotyping serves as the unifying clinical framework.

### 3.1. Lung Ultrasound as a Primary Imaging Tool in Neonatal Respiratory Failure

Lung ultrasound has progressively supplanted chest radiography as the first-line imaging modality in neonatal respiratory evaluation across leading neonatal intensive care units worldwide. Its advantages are particularly salient in the newborn: the neonatal thorax, characterized by a thin and compliant chest wall, unossified ribs, and high pulmonary water content, generates high-quality acoustic windows unavailable in older patients. This allows real-time, dynamic assessment of lung aeration, pleural integrity, and parenchymal pathology at the bedside, without radiation exposure or patient transport [9,10,15,16].

The acoustic signatures of common neonatal lung conditions are now well characterized. In RDS, bilateral diffuse B-lines—vertical laser-like artifacts arising from the pleural line—reflect alveolar–interstitial fluid and surfactant deficiency. B-line density and distribution form the basis of quantitative LUS scoring: the validated 0–18 point score, assigned across six lung zones (three per hemithorax), has demonstrated excellent predictive accuracy for surfactant requirement, with AUC values of 0.87 in late preterm and term infants and 0.854 in very preterm infants [11,12]. Cut-off values of ≤4 effectively exclude surfactant need, while scores ≥8 indicate high treatment probability, offering a simple, reproducible decision tool that transcends subjective clinical assessment [11,12].

Beyond the NICU, delivery room LUS has emerged as a powerful tool for early phenotyping of respiratory transition. A prospective monocentric study conducted in a Romanian tertiary referral center evaluated LUS patterns immediately after birth in term and near-term neonates. The study found that LUS aeration scores obtained within the first minutes of life accurately predicted subsequent respiratory support requirements, providing a clinically actionable framework for pre-emptive intervention—including early CPAP initiation or preparation for surfactant administration—before clinical deterioration occurs [9].

A quality improvement project implemented at a tertiary neonatal center in Bucharest demonstrated that a structured LUS protocol—performed at approximately 90 min after birth in all neonates requiring CPAP or mechanical ventilation—reduced chest radiograph use by more than 20%, without compromising diagnostic accuracy or clinical safety [10]. This Romanian experience aligns with international quality improvement data demonstrating that systematic POCUS implementation in the NICU is feasible, reproducible across operators with varying experience levels, and associated with meaningful reductions in ionizing radiation burden [16,17].

The ESPNIC POCUS Working Group has formalized the evidence base for neonatal LUS in a comprehensive international consensus, assigning Level B evidence to LUS for the diagnosis of RDS, transient tachypnea of the newborn (TTN), PTX, and pleural effusion, and for guidance of thoracentesis procedures [17]. These evidence grades reflect a maturing literature that has progressed from descriptive case series to prospective multicenter randomized trials evaluating LUS-guided surfactant protocols [11,12,18].

Table 1 summarizes the key LUS sonographic features relevant to the differential diagnosis of the most common neonatal respiratory conditions, including RDS, PH, PTX, TTN, and neonatal pneumonia, along with their respective LUS score implications and surfactant relevance.

### 3.2. Pulmonary Hemorrhage: Epidemiology, Ultrasonographic Phenotype, and Surfactant Rationale

Pulmonary hemorrhage in the preterm neonate is a life-threatening event characterized by the acute appearance of bloody or blood-tinged secretions from the endotracheal tube, accompanied by rapid deterioration in oxygenation and hemodynamic instability. PH generally manifests within the first 72 h of life and disproportionately affects very low birthweight (VLBW) infants with RDS. Global incidence estimates range from 1 to 12 per 1000 live births, with reported mortality in VLBW cohorts of 11–12%, rising to 50% or more in some series [19,20]. A cohort from Botswana documented a PH incidence of 4% (54/1350 hospitalized infants) with a case fatality rate of 53.7% [21].

Extreme prematurity constitutes the strongest independent risk factor, with capillary fragility and structural immaturity of the alveolar-capillary membrane rendering the pulmonary microvasculature susceptible to rupture under physiological stress. A national South Korean ELBW registry reported a PH incidence of 11.5% with attributable mortality of 20.5% in infants ≤25 + 2 weeks gestation with 5-min Apgar scores ≤2 [21]. Multivariate analyses identify hemodynamically significant patent ductus arteriosus (hsPDA), small for gestational age status, multiple gestation, elevated CRIB-II score, and prior surfactant use as independent predictors of massive PH [22]. The pathophysiological mechanism is increasingly understood as a consequence of left-to-right ductal shunting causing pulmonary venous hypertension and capillary overload, culminating in transudation and hemorrhage [20,22].

From an ultrasonographic perspective, PH generates distinctive and rapidly evolving LUS patterns that differ meaningfully from those of uncomplicated RDS, enabling bedside differentiation. The acute phase of PH is characterized by dense bilateral consolidation with a “tissue-like” echotexture, representing alveolar flooding with blood and proteinaceous fluid. Dynamic air bronchograms—mobile hyperechoic streaks within consolidated lung tissue—are often visible and reflect partial preservation of airway patency despite severe alveolar involvement. Shred signs, consisting of irregular, jagged deep margins to areas of consolidation, reflect the heterogeneous lung injury pattern. The pleural line becomes thick and irregular, and bilateral pleural effusions may be detectable. Crucially, B-lines in PH differ from those in RDS: they tend to be confluent, forming a “white lung” pattern bilaterally, and arise against a background of consolidated rather than simply atelectatic lung tissue [9,13].

Serial LUS examination provides objective monitoring of treatment response following surfactant administration—a feature unavailable with conventional radiography. Restoration of lung aeration is reflected by progressive reduction in consolidation depth, fragmentation of previously dense echotexture, and reappearance of B-lines from previously consolidated zones. Complete or near-complete resolution of the white-lung pattern within 6–12 h of rescue surfactant is a favorable prognostic sign [9,10].

The biological rationale for surfactant administration in PH derives from the documented inactivation of endogenous surfactant by hemoglobin, fibrin, and plasma proteins flooding the alveolar space. This inactivation disrupts the phospholipid film at the air-liquid interface, increasing surface tension, promoting diffuse atelectasis, increasing intrapulmonary right-to-left shunt, and driving refractory hypoxemia with escalating ventilatory pressure requirements [23,24,25]. Exogenous surfactant supplementation aims to reconstitute the surface-active film and restore alveolar compliance. Case series and retrospective cohort data support transient but clinically meaningful improvements in oxygenation and ventilatory parameters following surfactant administration in stabilized patients [25,26,27,28].

However, no randomized controlled trial (RCT) has evaluated surfactant specifically in neonatal PH, and the absence of high-quality evidence limits definitive recommendations. Available data derive from retrospective series, observational studies, and expert consensus, all subject to substantial confounding by disease severity. A Cochrane review by Aziz and Ohlsson (2020) concluded that current evidence is insufficient to confirm or refute the efficacy of surfactant in neonatal PH, and called for prospective trials [25]. A multinational survey of 360 clinicians revealed highly variable management practices, with most using post-event surfactant administration alongside frequent hsPDA evaluation, but without standardized protocols [29].

Importantly, the relationship between surfactant therapy and PH incidence is bidirectional. Several studies identify surfactant administration as an independent risk factor for subsequent PH, likely by rapidly lowering pulmonary vascular resistance and augmenting left-to-right ductal shunting in the presence of hsPDA, thereby increasing pulmonary blood flow and capillary stress [30,31,32]. Multivariate analyses indicate that this association primarily reflects disease severity rather than a direct causal effect of surfactant [22]. The 2024 Turkish retrospective study by İyigün et al. identified distinct early (≤72 h) and late (>72 h) PH phenotypes: early PH was associated with higher surfactant requirements, while late PH was linked to sepsis, coagulopathy, and hsPDA—both phenotypes with similar final outcomes [33].

Current expert guidance recommends that surfactant administration in PH be deferred until after initial stabilization, with mandatory hemodynamic assessment—including functional echocardiography for ductal status and pulmonary vascular resistance estimation—prior to dosing. The LUS phenotype at the time of potential surfactant administration should guide decision-making: persistent white-lung consolidation with absent surfactant response in ventilatory parameters, in a hemodynamically optimized neonate, constitutes the most defensible indication. Repeat dosing should follow the same LUS-guided criteria applied in RDS redosing protocols [12,28,29].

### 3.3. Pneumothorax: LUS-Based Diagnosis, Risk Stratification, and Therapeutic Sequencing

Neonatal pneumothorax represents the pathological accumulation of free air in the pleural space and spans a clinical spectrum from asymptomatic, self-resolving forms to rapidly fatal tension PTX with cardiorespiratory collapse. Its multifactorial etiology encompasses the physiological stresses of birth transition, airway obstruction, underlying parenchymal lung disease (RDS, meconium aspiration syndrome, pneumonia), and iatrogenic barotrauma from invasive or non-invasive ventilatory support [34,35].

Epidemiological data from a large multicenter cohort of approximately 58,700 infants documents an overall PTX prevalence of 0.53%, rising to 4.0% in infants born at 29–34 weeks and 4.6% in those ≤28 weeks. Most cases present within the first 24 h, particularly in infants ≥35 weeks (76%), where the physiological challenges of respiratory transition predominate. Delivery room respiratory interventions—CPAP initiation and endotracheal intubation—are stronger determinants of PTX risk than NICU-administered bubble CPAP, highlighting the importance of standardized delivery room protocols [34]. Incidence within the Swiss perinatal network ranged from 0.05% to 2%, reflecting significant inter-center variability in patient populations, diagnostic criteria, and management approaches [35].

Lung ultrasound has established itself as the gold standard for bedside PTX diagnosis in the neonate, demonstrating superior sensitivity and specificity compared to chest radiography, with the added advantage of immediacy, portability, and repeatability without radiation exposure [15]. The international expert consensus published in 2020 by Liu et al. established standardized sonographic criteria and ultrasound-guided intervention protocols for neonatal PTX [17]. The hallmark LUS findings are: (1) absent lung sliding—the absence of the characteristic shimmering motion at the pleural interface; (2) absent B-lines—reflecting interposition of air between the probe and aerated lung; (3) the lung point—the pathognomonic transition between the air-filled pleural space and normally aerated lung, serving as the precise anatomical guide for thoracentesis needle placement; and (4) the stratosphere sign on M-mode, replacing the normal seashore sign [15,36].

The diagnostic algorithm for suspected PTX in the NICU should begin with immediate LUS evaluation. Confident identification of the lung point not only confirms the diagnosis but lateralizes the lesion and delineates its extent, informing the urgency and modality of drainage—needle aspiration versus intercostal drain placement [37,38]. A comparison of intervention strategies from a Cochrane review demonstrated that needle aspiration, while less invasive, carries higher failure rates than drain placement, necessitating careful patient selection and close LUS surveillance post-procedure [37]. In tension PTX, LUS-guided needle decompression can be life-saving in the delivery room or during transport, precluding the delay inherent to radiographic confirmation [12,15].

The relationship between surfactant therapy and PTX is primarily mediated by underlying RDS severity and ventilatory pressure requirements. Early, guideline-consistent surfactant administration—particularly via minimally invasive techniques—reduces the need for high-pressure mechanical ventilation, thereby lowering the risk of air leak syndromes [39,40,41]. Conversely, non-standardized or delayed surfactant use, associated with greater disease severity and higher ventilatory pressures, correlates with increased PTX rates—a relationship that reflects severity confounding rather than causation [42]. In neonates with established PTX, LUS after drainage confirms adequate lung re-expansion and identifies residual or contralateral air leak requiring further intervention. If underlying RDS is confirmed by LUS following PTX resolution, surfactant administration is indicated using standard dosing criteria, with careful post-instillation monitoring given the altered compliance dynamics [26,39,43].

### 3.4. Pulmonary Surfactant: Biology, Preparations, and Clinical Pharmacology

Pulmonary surfactant is a complex lipoprotein mixture synthesized and secreted by type II alveolar pneumocytes. Composed predominantly of phosphatidylcholine (approximately 70% of phospholipid content), with smaller proportions of phosphatidylglycerol and other lipids, and critically supplemented by hydrophobic proteins SP-B and SP-C, surfactant reduces surface tension at the air-liquid interface during expiration, preventing alveolar collapse at end-expiration and enabling efficient gas exchange [23,24,44]. Beyond its biophysical role, surfactant participates in innate pulmonary immunity, modulating inflammatory signaling and facilitating pathogen clearance [24].

The first successful clinical use of exogenous surfactant, reported by Fujiwara in 1980, initiated a therapeutic revolution in neonatology [45]. Subsequent large multicenter randomized trials—including the OSIRIS trial and multiple European studies—established that animal-derived natural surfactants significantly improved oxygenation, reduced air-leak syndromes, and lowered neonatal mortality compared to placebo and early synthetic preparations [46,47]. Comparative studies confirmed the superiority of natural surfactants (poractant alfa, beractant, calfactant) over early synthetic formulations in terms of rapidity of response and clinical outcomes [47,48].

In the preterm lung, insufficient surfactant production and delayed secretion predispose to alveolar instability, progressive atelectasis, and ventilation-perfusion mismatch. Preservation of native surfactant function through lung-protective ventilation strategies is therefore a key determinant of respiratory outcome and underpins the rationale for non-invasive respiratory support [44]. Current evidence-based guidelines recommend early rescue surfactant administration in preterm infants with RDS rather than delayed treatment, with threshold FiO_2_ values of approximately 0.30–0.50 on CPAP with PEEP ~7 cmH_2_O representing pragmatic intervention triggers [26,39,49].

Table 2 summarizes the principal surfactant preparations in current clinical use, with their recommended dosing regimens, biological origins, and administration characteristics.

### 3.5. LUS-Guided Surfactant Strategies: Scoring, Administration Techniques, and Comorbidity Interactions

The integration of quantitative LUS scoring into surfactant decision pathways has transformed clinical practice from a threshold-based empiricism to a phenotype-driven precision approach. Classical criteria—FiO_2_ requirements, ventilatory pressure parameters—remain clinically relevant, but are substantially augmented by LUS-derived aeration scores that capture real-time lung condition with reproducible objectivity [11,12,18,39].

A prospective international multicenter study (2022–2023) evaluating infants ≥34 weeks with early respiratory failure demonstrated that LUS score predicted surfactant requirement with an AUC of 0.87; scores >8 identified high treatment likelihood while scores ≤4 reliably excluded surfactant need, with 95% negative predictive value [11]. A subsequent multicenter study of infants ≤30 weeks applied LUS scoring at approximately 12 h of life (≥10 h post-initial dosing) to predict re-dosing requirements, achieving AUC 0.854, with a threshold of 4 yielding 98% sensitivity and threshold of 8 providing 78% overall accuracy, consistently across gestational age subgroups [12]. These data establish LUS score thresholds of 4 and 8 as pragmatic, reproducible clinical decision anchors for both initial dosing and re-dosing in RDS, and by extension, in PH where the scoring system captures the alveolar flooding pattern [11,12,55,56].

The LUNG study (Lung UltrasouNd Guided surfactant therapy)—an ongoing international multicenter randomized controlled trial—is evaluating whether LUS-guided surfactant administration in preterm infants outperforms conventional criteria in terms of reducing mechanical ventilation exposure and respiratory morbidity [18]. Interim and observational evidence already supports LUS-based surveillance for extubation readiness, re-dosing timing, and long-term morbidity prediction, including bronchopulmonary dysplasia risk stratification from early LUS scores [37,57,58,59].

The method of surfactant delivery has evolved substantially. Over the past decade, less invasive surfactant administration (LISA)—delivery via thin catheter or similar device during spontaneous breathing on CPAP, without endotracheal intubation—has accumulated compelling evidence from multiple RCTs including the AMV, TakeCare, NINSAPP, and OPTIMIST-A trials [26,60,61,62]. These studies demonstrate consistent reductions in mechanical ventilation exposure and improvement in composite respiratory outcomes compared to INSURE (Intubate–Surfactant–Extubate), while avoiding the hemodynamic perturbations associated with positive-pressure ventilation. European, British, and Canadian guidelines now identify LISA as the preferred delivery approach in spontaneously breathing preterm infants on CPAP [39,63,64,65].

In the context of PH and PTX, the choice of administration technique requires careful individualization. In PH, the immediate post-hemorrhagic period is characterized by hemodynamic instability and coagulopathy; surfactant should be administered only after hemodynamic stabilization is confirmed—including echocardiographic assessment of ductal status and pulmonary vascular resistance—and when LUS documents persistent consolidation unresponsive to ventilatory optimization. The post-instillation period requires vigilance: rapid restoration of compliance can precipitate barotrauma if ventilatory pressures are not promptly reduced. LUS in the minutes to hours following surfactant allows real-time assessment of re-aeration and guides pressure weaning [25,26,28,66].

In PTX, the therapeutic sequence is invariable: drainage precedes surfactant consideration. Once pleural air is evacuated and LUS confirms effective lung re-expansion, surfactant is indicated if LUS demonstrates residual RDS pattern (diffuse B-lines, bilateral consolidations) contributing to ongoing respiratory failure. Laryngeal mask airway (LMA)-mediated surfactant delivery has been reported in neonates with RDS and coexisting PTX as a strategy to avoid intubation and re-inflation pressures, though generalization of this approach requires further validation [43,67].

Comorbidities—hsPDA, persistent pulmonary hypertension of the newborn (PPHN), sepsis, and coagulopathy—significantly modify surfactant response in PH and PTX. Hemodynamically significant PDA amplifies pulmonary overcirculation, increases capillary stress, and promotes alveolar protein flooding that inactivates administered surfactant. Echocardiographic ductal assessment is therefore an integral component of pre-surfactant evaluation in PH, and pharmacological or interventional ductal closure should be considered when feasible [30,32,68,69]. Sepsis-associated inflammatory mediators further suppress surfactant synthesis and promote protein-mediated inactivation, creating a vicious cycle of refractory respiratory failure that may require escalating doses and repeated LUS reassessment [33,70].

Table 3 provides a comprehensive summary of the key clinical studies included in this review, detailing study characteristics, sample sizes, comorbidities, and main findings, offering a synthesized evidence matrix for clinical reference.

## 4. Discussion

The clinical management of neonatal PH and PTX is evolving from a reactive, empirical discipline toward a proactive, ultrasonographically-informed precision medicine approach. The central argument of this review is that LUS is not merely a diagnostic adjunct in these conditions, but rather the primary decision architecture—the tool that defines when, whether, and how surfactant should be administered, and how the therapeutic response should be monitored. This reframing has significant implications for clinical protocol design, NICU infrastructure, and training priorities.

The pathophysiological complexity underlying surfactant failure in PH and PTX is well characterized [23,24,25,74,75]. In PH, alveolar flooding with blood and plasma proteins irreversibly inactivates endogenous surfactant, dismantling the protective phospholipid film. Exogenous surfactant supplementation may transiently restore compliance and oxygenation, but only if administered after hemodynamic optimization—particularly ductal assessment and management—and only when LUS confirms a persistent consolidation-dominated phenotype amenable to surfactant-mediated re-aeration [25,27,28,30]. The 2020 Cochrane review by Aziz and Ohlsson remains authoritative in acknowledging the biological plausibility of surfactant rescue in PH while simultaneously underscoring the absence of RCT evidence [25]. This evidence gap should motivate, not paralyze, clinical decision-making: when LUS demonstrates a white-lung pattern, physiological principles support a trial of rescue surfactant in a hemodynamically stable, coagulopathy-managed neonate.

The role of hsPDA in modulating PH risk and surfactant response deserves particular emphasis. Multiple studies confirm that left-to-right ductal shunting elevates pulmonary venous pressure, promotes capillary transudation, and amplifies the protein load that inactivates both endogenous and exogenous surfactant [20,30,32,68]. Functional echocardiography, when performed alongside LUS, provides the hemodynamic phenotype necessary to individualize surfactant timing and minimize the risk of paradoxical worsening through pulmonary overcirculation. This integrated POCUS assessment—combining LUS for parenchymal phenotype and echo for hemodynamic context—represents the clinical gold standard for surfactant decision-making in PH [69,70,76].

In PTX, the primacy of LUS is even more established. The international consensus by Liu et al. (2020) has standardized sonographic diagnostic criteria and ultrasound-guided thoracentesis techniques for neonatal PTX, establishing a clear procedural framework [15]. The clinical burden of delayed diagnosis—particularly in tension PTX—is mitigated by LUS-equipped teams capable of diagnosis and drainage within seconds of clinical suspicion. A comparison of drainage modalities confirms that while needle aspiration is less invasive, drain placement achieves more reliable resolution; LUS guides both the choice and the execution of the procedure [37,38]. The relationship between surfactant and PTX is primarily confounded by RDS severity: early, guideline-adherent surfactant delivery via LISA reduces barotrauma exposure and PTX incidence, while delayed or high-pressure administration correlates with greater air-leak risk—a distinction critical for NICU quality improvement efforts [40,41,42,73].

The evidence base for quantitative LUS scoring in surfactant decision-making has matured substantially. The validated thresholds of 4 and 8 on the 0–18 point scoring system provide reproducible clinical anchors across gestational age groups, and their integration into redosing protocols offers a standardized, objective alternative to the heterogeneous clinician-dependent criteria currently in use [11,12,37,57]. Although these scores have demonstrated clinical utility in evaluating neonatal respiratory distress and guiding respiratory support, interobserver variability remains an important consideration, particularly in borderline or mixed-pattern examinations. Standardized scanning protocols, structured operator training, and the increasing integration of computer-assisted image analysis may improve reproducibility and facilitate broader implementation in neonatal intensive care settings. From a practical perspective, simplified clinical algorithms integrating LUS findings with respiratory status may support timely bedside decision-making and optimization of neonatal respiratory management. The prospective multicenter LUNG trial, currently underway, will provide the highest-quality evidence to date on LUS-guided surfactant protocols in preterm RDS, with implications that will extend to the management of PH and PTX [18].

National-level implementation of LUS protocols in NICUs, as demonstrated by the Romanian quality improvement experience [9,10], underscores that the barriers to adoption are primarily organizational rather than technological. Structured training programs, validated competency assessment tools, and institutional protocol development are the key enablers. The ESPNIC POCUS guidelines provide a robust evidence-based framework for this implementation [17], and neonatal POCUS networks—including nationally coordinated initiatives—represent the most efficient pathway toward standardized, equitable LUS access across neonatal units of varying resource levels [12,16].

Table 4 summarizes the key take-home messages regarding the use of lung ultrasound (LUS) in neonatal intensive care practice, highlighting its diagnostic role, therapeutic implications, and practical impact on bedside decision.

The main limitation of this review is its narrative design, which precludes formal risk-of-bias assessment and quantitative meta-analysis. The included evidence is heterogeneous in design, population, and endpoint definition. For PH specifically, the absence of RCTs constitutes a fundamental evidentiary limitation, and the observational literature is uniformly confounded by severity bias—surfactant use marks sicker infants, making causal inference about its efficacy in PH inherently difficult [77]. For PTX, large epidemiological cohorts and case series provide robust prevalence estimates but limited standardization of management protocols [29,35]. Emerging approaches—LUS-guided management, LISA in complex scenarios, LMA-mediated surfactant delivery—remain insufficiently validated by long-term outcome data, though the physiological rationale and early clinical evidence are encouraging [26,43,67].

The parallel importance of early neurodevelopmental surveillance in neonates who survive PH and PTX must be acknowledged. High-quality neonatal care and optimized respiratory management have long-term implications beyond the neonatal period. Standardized neurological assessment frameworks—including general movements assessment and structured cranial ultrasound protocols—are essential components of holistic neonatal care [78,79,80].

Pulmonary echography, particularly lung ultrasound (LUS), extends beyond acute diagnosis to support continuous neonatal respiratory monitoring and early respiratory stabilization. Despite limitations related to artifact interpretation, operator dependency, and standardization, advances in computational image analysis and pattern recognition may improve reproducibility and expand its role within integrated neonatal care frameworks. In this context, LUS represents a valuable component of multimodal neonatal management linking early respiratory stability with long-term outcomes [81,82]. International experience in coordinated chronic disease management and structured care pathways reinforces the value of integrated, protocol-driven approaches across the full continuum of neonatal and pediatric care [83,84].

Prospective multicenter RCTs are urgently needed to define optimal surfactant indications, dosing, timing, and re-dosing criteria in neonatal PH, with LUS-based stratification as a foundational design element. For PTX, standardization of LUS-guided drainage protocols and post-PTX surfactant criteria across NICU networks would substantially reduce the inter-center variability currently documented in the literature.

## 5. Conclusions

Neonatal pulmonary hemorrhage and pneumothorax represent acute, life-threatening complications in preterm and high-risk infants, characterized by high morbidity and mortality despite the significant advances of modern neonatal care. Exogenous surfactant therapy, firmly established as the cornerstone of RDS management, occupies an uncertain yet physiologically rational role in both PH and PTX, with current use guided by clinical judgment, pathophysiological principles, and—increasingly—ultrasonographic phenotyping.

Lung ultrasound is the integrating tool that makes individualized surfactant decision-making feasible at the bedside. Validated quantitative LUS scoring systems provide reproducible, objective criteria for initial dosing and re-dosing that outperform empirical threshold-based approaches. Delivery room LUS enables pre-emptive identification of at-risk neonates; serial post-surfactant LUS enables real-time therapeutic monitoring. Nationally implemented LUS protocols demonstrate meaningful reductions in radiation exposure without clinical compromise. The ESPNIC consensus provides the evidence-based framework for POCUS integration into neonatal respiratory care.

A LUS-anchored, physiology-driven algorithm—combining ultrasonographic phenotyping, hemodynamic assessment via functional echocardiography, and judicious application of minimally invasive surfactant techniques—represents the current best standard of care for these vulnerable populations. Standardization of POCUS training, protocol implementation, and inter-center benchmarking are the most pressing near-term priorities. Randomized controlled trials specifically designed around LUS-guided surfactant strategies in PH and PTX are the critical evidence gap that the neonatal community must urgently address.

## Figures and Tables

**Table 1:** Lung ultrasound (LUS) sonographic features in neonatal respiratory conditions: diagnostic differentiation and surfactant relevance.

LUS Feature	RDS	Pulmonary Hemorrhage	Pneumothorax	TTN/Pneumonia
Lung sliding	Present	Reduced/absent over consolidated zones	Absent (pathognomonic)	Present
B-lines	Diffuse, bilateral, homogeneous	Diffuse, fused; alveolar flooding pattern	Absent	TTN: bilateral comet tails; Pneumonia: focal
Consolidation	Absent or minimal (atelectasis)	Tissue-like; shred sign; dynamic air bronchograms	Absent	Pneumonia: subpleural/lobar consolidation
Lung point	Absent	Absent	Present (pathognomonic; guides thoracentesis)	Absent
Pleural line	Irregular; subpleural consolidations	Irregular, thickened; possible effusion	No sliding; stratosphere sign (M-mode)	TTN: regular; Pneumonia: focal irregularity
LUS Score (0–18)	≥8: high surfactant need; ≤4: unlikely needed	High (≥8–12); guides rescue surfactant timing	N/A—diagnosis by absence of sliding/B-lines	TTN: moderate; typically resolves without surfactant
Surfactant implication	Primary indication; LUS-guided dosing	Rescue after hemodynamic stabilization; serial LUS monitoring	Post-drainage; confirm RDS by LUS before dosing	Generally not indicated

RDS: respiratory distress syndrome; TTN: transient tachypnea of the newborn; LUS: lung ultrasound; N/A: not applicable. Adapted from [9,10,11,12,13,14,15,17].

**Table 2:** Dosing regimens, administration characteristics, and biological origin of commonly used surfactant preparations.

Surfactant	Origin	Typical Dose (mg/kg)	Number of Doses	Day of Administration
Poractant alfa [49]	Porcine	200 mg/kg initial; 100 mg/kg repeat	1–3 doses	Day 0 (within hours of birth); repeats Day 1–2
Beractant [50]	Bovine	100 mg/kg per dose	Up to 4 doses (~6 h intervals)	Day 0–1 early rescue; may continue to Day 2
Calfactant [51]	Bovine	105 mg/kg per dose	Up to 3 doses (~12 h intervals)	Day 0–1, occasionally Day 2
Lucinactant [52]	Synthetic	175 mg/kg per dose	Usually 1–2 doses	Day 0 (prophylaxis or early rescue)
Bovactant [53]	Bovine	50 mg/kg initial; variable repeats	1–2 doses	Day 0–1

Dosing based on product-specific guidelines [50,51,52,53,54].

**Table 3:** Summary of key studies on surfactant therapy in neonatal pulmonary hemorrhage and pneumothorax: design, comorbidities, and principal findings.

Authors [Ref]	Year	Country	Study Design	N	Comorbidities	Main Findings
Ren et al. [71]	2020	China	Systematic Review	7 studies	RDS	Decreased mortality and adverse events with surfactant in pediatric RDS
Mani et al. [8]	2024	USA	Narrative Review	N/A	RDS, PH	Surfactant recommended in RDS; direct effect on pulmonary hypertension outcomes remains uncertain
Wang et al. [13]	2024	China	Retrospective	5548	RDS, MAS	Surfactant used in 38.5%; increasing use in MAS
Li et al. [20]	2021	China	Retrospective	499	PH, hsPDA, coagulopathy	PDA + coagulopathy → ~70% massive PH risk
Jung et al. [22]	2024	Republic of Korea	Retrospective	13,826	RDS, PH, hsPDA	Independent massive PH risk factors: SGA, multiple gestation, high CRIB-II, surfactant use, hsPDA
Farghaly et al. [34]	2025	USA	Retrospective	58,706	PTX	CPAP prevents intubation; improper use may increase air leak risk
Aziz & Ohlsson [25]	2020	Canada	Cochrane Review	N/A	RDS, PH	No RCT evidence supports surfactant in neonatal PH; further trials warranted
Dargaville et al. [26]	2023	Australia	Review	N/A	RDS, MAS, PH	Surfactant essential for RDS; only transient oxygenation benefits in MAS and PH
Sweet et al. [39]	2022	International	Consensus Guidelines	N/A	RDS, PTX	Early surfactant for worsening RDS; LISA preferred; repeat dosing guided by clinical response
De Luca et al. [11]	2023	International	Prospective Multicenter	≥34 wk cohort	RDS	LUS score predicted surfactant need: AUC 0.87; scores >8 indicate high likelihood; ≤4 rules out
Jagła et al. [12]	2025	Multicenter	Prospective Multicenter	≤30 wk cohort	RDS	LUS AUC 0.854 for re-dosing; threshold 4 → 98% sensitivity; threshold 8 → 78% accuracy
Dargaville et al. [72]	2021	International	RCT (OPTIMIST-A)	486	RDS, PTX	MIST reduces death or BPD vs. sham in preterm infants on CPAP
Barnes et al. [28]	2022	Ireland	Systematic Review	16	PH	Surfactant improves oxygenation and ventilatory indices in PH; limited evidence; no clear mortality impact
Thakkar et al. [29]	2024	International	Global Survey	360 replies	PH, hsPDA	PH management highly variable; most use post-event surfactant; no standardized guidelines
Heiring et al. [73]	2025	Denmark	Retrospective	108	RDS, PTX, PH, hsPDA	LISA reduces mechanical ventilation; no differences in PH, PTX, hsPDA, or mortality rates
Sahussarungsi et al. [30]	2025	Canada	Review	N/A	RDS, PH, hsPDA	Surfactant improves RDS outcomes; in preterm infants with hsPDA may increase PH risk via hemodynamic changes
İyigün et al. [33]	2026	Turkey	Retrospective	N/A	RDS, PH, hsPDA	Early PH (≤72 h): higher surfactant need; late PH: sepsis/coagulopathy/hsPDA dominant; similar outcomes
Toma et al. [9]	2024	Romania	Prospective Monocentric	N/A	RDS, TTN	Delivery room LUS predicts respiratory support need; integration into NICU protocols feasible
Nemes, Toma, Dima et al. [10]	2024	Romania	Quality Improvement Project	125	RDS	LUS protocol reduces chest X-ray use by >20% without compromising diagnostic accuracy or safety

RDS: respiratory distress syndrome; PH: pulmonary hemorrhage; PTX: pneumothorax; CRIB-II: clinical risk index for babies II; CPAP-continuos positive airway pressure; hsPDA: hemodynamically significant patent ductus arteriosus; MAS: meconium aspiration syndrome; TTN: transient tachypnea of the newborn; NICU-neonatal intensive care unit; BPD: bronchopulmonary dysplasia; MIST: minimally invasive surfactant therapy; LISA: less invasive surfactant administration; LUS: lung ultrasound; AUC: area under the curve; N/A: not applicable; RCT: randomized controlled trial. Only a representative selection of included studies is shown.

**Table 4:** Key Take-Home Messages for the Use of LUS in Neonatal Intensive Care Practice.

	LUS Findings & Diagnostics	Therapeutic Implications & Management	Practical Clinical Impact
1. Ultrasound as First-Line (POCUS)	Replaces conventional chest X-rays (CXR). Real-time, dynamic bedside evaluation.	Eliminates diagnostic delays in the NICU. Allows for safe, serial follow-up scans.	Reduces neonatal radiation exposure by >20% without compromising diagnostic accuracy or safety.
2. Quantitative LUS Score (0–18)	Score 4: Good lung aeration. Score 8: Severe alveolar collapse or fluid flooding.	Score 4: Excludes surfactant need. Score 8: Strongly indicates initial dosing or re-dosing.	Replaces subjective decisions based on arbitrary FiO_2_ thresholds. Excellent predictive accuracy (AUC 0.85–0.87).
3. Pulmonary Hemorrhage (PH)	Reveals dense, “tissue-like” consolidations, shred sign, diffuse B-lines, and alveolar flooding patterns.	Blood destroys endogenous surfactant. Rescue surfactant must be deferred until after hemodynamic stabilization and ductal (hsPDA) echo assessment.	Prevents paradoxical worsening of left-to-right shunts while rapidly restoring surfactant film compliance.
4. Pneumothorax (PTX)	Pathognomonic triad: absent lung sliding absent B-lines, and a visible lung point.	Clinical sequence is strict: air drainage always precedes surfactant administration.	Provides exact anatomical guidance for needle thoracentesis, avoiding blind punctures or waiting for a CXR.
5. Minimally Invasive Techniques	Real-time tracking of lung re-aeration changes during non-invasive respiratory support.	Strongly supports the use of LISA in spontaneously breathing infants.	The LUS-LISA synergy minimizes mechanical ventilation exposure, intubation rates, and ventilator-induced lung injury.
6. Precision Neonatology	Multimodal POCUS approach: lung ultrasound phenotyping with functional echocardiography.	Allows for real-time individualization of airway pressure weaning based on objective structural responses.	Mitigates severe hypoxic episodes and fluctuations in cerebral blood flow, protecting long-term neurodevelopment.

Note: The structure and layout of this table were generated using AI.

## Data Availability

No new data were created or analyzed in this study.

## Abbreviations

The following abbreviations are used in this manuscript:

AUC	Area Under the Curve
BPD	Bronchopulmonary Dysplasia
CPAP	Continuous Positive Airway Pressure
CRIB-II	Clinical Risk Index for Babies II
ELBW	Extremely Low Birth Weight
ESPNIC	European Society of Paediatric and Neonatal Intensive Care
FiO_2_	Fraction of Inspired Oxygen
hsPDA	Hemodynamically Significant Patent Ductus Arteriosus
INSURE	Intubate–Surfactant–Extubate
LISA	Less Invasive Surfactant Administration
LMA	Laryngeal Mask Airway
LUS	Lung Ultrasound
MAS	Meconium Aspiration Syndrome
MIST	Minimally Invasive Surfactant Therapy
NICU	Neonatal Intensive Care Unit
PH	Pulmonary Hemorrhage
POCUS	Point-Of-Care Ultrasound
PPHN	Persistent Pulmonary Hypertension of the Newborn
PTX	Pneumothorax
RCT	Randomized Controlled Trial
RDS	Respiratory Distress Syndrome
SGA	Small for Gestational Age
TTN	Transient Tachypnea of the Newborn
VLBW	Very Low Birth Weight
